# Prediction of fear of falling at 6 months after stroke based on 279 individuals from the Fall Study of Gothenburg

**DOI:** 10.1038/s41598-021-92546-9

**Published:** 2021-06-29

**Authors:** Netha Hussain, Per-Olof Hansson, Carina U. Persson

**Affiliations:** 1 Department of Occupational Therapy and Physiotherapy, Sahlgrenska University Hospital/Östra, Gothenburg, Region Västra Götaland Sweden; 2 Department of Molecular and Clinical Medicine, Sahlgrenska Academy, University of Gothenburg, Sweden and Sahlgrenska University Hospital, Gothenburg, Region Västra Götaland Sweden; 3 Department of Clinical Neuroscience, Rehabilitation Medicine, Institute of Neuroscience and Physiology, Sahlgrenska Academy, University of Gothenburg, Gothenburg, Region Västra Götaland Sweden

**Keywords:** Neuroscience, Neurology

## Abstract

The early identification of individuals at risk of fear of falling after stroke is crucial in order to individualise preventive actions and interventions. The aim of this study was to identify the incidence of, and baseline factors in acute stroke that are associated with fear of falling at 6 months after stroke. Fear of falling was assessed by one question, which was answered by 279 of 452 eligible individuals. Univariable and multivariable logistic regression analyses were performed to determine the factors that were associated with fear of falling. The dependent variable was fear of falling at 6 months after stroke. The independent variables were related to function, activity and participation, including personal and environmental factors. Fear of falling was reported by 117 (41.9%) individuals. Poor postural control in acute stroke, measured using the modified version of the Postural Assessment Scale for Stroke Patients (odds ratio [OR]: 2.60, 95% confidence interval [CI]: 1.26–5.36), and being physically inactive prior to the stroke, measured using the Saltin-Grimby Physical Activity Scale (OR: 2.04, 95% CI: 1.01–4.12), were found to be associated with fear of falling at 6 months after stroke. The findings in this study are useful in clinical practice to optimise rehabilitation after stroke.

## Introduction

Fear of falling is a psychological condition associated with excessive worrying about losing one’s balance^1^. Individuals with fear of falling worry about the physical harm and consequent lasting functional disability which could occur as a result of falling^2^. They are also concerned about the social embarrassment and indignity associated with falling^2^. Fear of falling may lead to the avoidance of engaging in day-to-day activities^3,4^ as well as adoption of overly cautious behaviours^5,6^, in addition to reduced physical function, isolation and poorer quality of life^7–9^.

One of the leading causes of disability worldwide is stroke^10,11^. Individuals with stroke are more likely to experience fear of falling compared with those without a stroke^12–14^. As stroke onset is acute, cognitive and bodily changes occur instantly, leaving people highly vulnerable to fear of falling^7^. Due to the differences between the development and progress of fear of falling in different health conditions, research in this area should focus on fear of falling in specific disease conditions, such as stroke.

Previous studies show that up to 51% of inpatients^15^, 59% after hospital discharge^16^ and 48% of community-dwelling people^17^ report fear of falling after stroke. At 6 months after stroke, when individuals are likely to have adapted their behaviour to reduce falling to some extent, more than 50% of the individuals reported fear of falling after stroke^18,19^. A small qualitative study showed that individuals after stroke continue to report fear of falling as late as 7 years after stroke and require physical and cognitive adjustment to combat their fear of falling^20^. Due to its widespread prevalence and long-lasting consequences, it is important to address the issue of fear of falling in individuals with stroke.

The early identification of individuals at risk of fear of falling is crucial in order to prevent its occurrence and optimise the rehabilitation after stroke. In the late subacute phase of stroke, fear of falling was found to be associated with low muscle strength in the lower limbs^18^. A previous study from the same cohort as in the current study, has shown that female sex, the use of a walking aid and poor postural control are risk factors for fear of falling in the acute phase, when assessed within one to seven days after stroke^21,22^. Findings from a few studies in the chronic phase of stroke (more than 6 months after stroke onset)^22^ show that greater fear of falling was associated with higher age, history of past falls, lower functional mobility, reduced activity, increased anxiety or depression, impaired postural control, high sitting time and worse stroke severity^12,23–25^.

A few studies^7,18,26^ have documented fear of falling in the late subacute phase of stroke, at three to 6 months after stroke onset^22^. One study found that fear of falling was associated with low muscle strength in the lower limbs in the late subacute phase of stroke^18^. However, these studies have small sample sizes (n = 28–50)^7,18,26^ or deal specifically with the association of fear of falling with factors affecting quality of life^18,26^. The data relating to the risk factors for fear of falling during the late subacute phase of stroke are therefore sparse^22^, although individuals have frequently mentioned fear of falling as a major concern in this phase^27^.

The aim of this study was to identify the incidence of fear of falling, and the factors prior to a stroke and in the acute phase after a stroke that are associated with fear of falling at 6 months after a stroke. Our hypothesis was that fear of falling at 6 months after a stroke was associated particularly with female sex and poor postural control, along with factors such as previous fall(s) at the stroke unit, the use of a walking aid and self-reported physical inactivity.

## Methods

### Study design

This prospective, longitudinal cohort study is a six-month follow-up of ‘*The Fall Study of Gothenburg’* (FallsGOT)^15^. In FallsGOT, a consecutive sample of 504 individuals 18 years or older, with a clinical diagnosis of stroke and admitted to the stroke unit at Sahlgrenska University Hospital/Östra in Gothenburg between 1 October 2014 and 30 June 2016 was studied. The cohort did not include individuals who were judged as suitable to receive thrombolysis or thrombectomy, as they were referred to another stroke unit in the city. The exclusion criteria were: unwillingness to participate in the study, those patients undergoing palliative care or those with severe dementia. The participants in the FallsGOT cohort have been described in detail in previous studies^15,21,28^. All the participants in the FallsGOT cohort who were still alive 6 months after a stroke were invited to participate via a postal questionnaire. The sample size analyses for the study cohort (original sample size = 504) have previously been presented^15^.

### Clinical assessments of the independent variables at baseline

All the assessments were made by the staff at the stroke unit. Data on age, sex, height, weight, location, type of stroke and length of stay at the hospital were collected from medical records. The subtyping of ischemic strokes based on presenting symptoms and signs was performed using the Oxford classification system^29^.

Information about falls was collected by the nurses at the stroke unit according to clinical routine. A fall was defined according to the World Health Organisation (WHO) as ‘an event which results in a person coming to rest inadvertently on the ground or floor or other lower level’^30^. The definition was given to the members of the stroke unit prior to the data collection stage. Stroke severity was assessed using the National Institute of Health Stroke Scale (NIHSS)^31^. The NIHSS scores were classified into mild (0–4 points), moderate (5–15 points) and severe stroke (16–42 points)^32^. Cognitive function was assessed by the occupational therapist using the Montreal Cognitive Assessment (MoCA) scale, which is an 11-item ordinal scale with a total score ranging from 0 to 30, which was performed for all inpatients with stroke sometime during the care period^33^. A higher score on the MoCA scale indicates better cognitive function and a score of 26 or higher indicates normal cognitive function^34^. Fear of falling during the acute phase of stroke was assessed by asking ‘Are you afraid of falling?’ with yes and no as possible answers.

The standardised assessment of the individual’s activity capacity of postural control was performed by the physiotherapists at the stroke unit using the Modified Version of the Postural Assessment Scale for Stroke Patients (SwePASS). In SwePASS, postural control refers to the ability to maintain a given posture in lying, sitting and standing and to ensure equilibrium in changes of position related to different levels of support and timing. The SwePASS is a 12-item ordinal clinical scale with four response categories per item and a total score ranging from 0 to 36^35,36^. It has been shown to have good intra-rater and inter-rater reliability^37–39^, as well as good responsiveness^40^. The SwePASS has also been found to be a moderate predictor of falls during the first year after stroke^28^, a strong predictor of falls during the stay at the stroke unit^15^ and within the first 12 months after stroke onset^28^ as well as of recurrent falls within the first year post-stroke^41^. The SwePASS scores were categorised as poor (0–24 points), moderate (25–30) or good (31–36) postural control based on previous evidence on its ability to predict falls^15^. Self-perceived physical activity prior to the stroke event was estimated using the Saltin-Grimby Physical Activity Level Scale (SGPALS)^42^. The SGPALS is a one-item self-reported outcome measurement including four response categories. A higher score represents a higher self-reported physical activity level. An SGPALS score of 1 was regarded as physically inactive and scores between 2 and 4 were regarded as light to high physical activity.

All assessments, apart from the MoCA, were made as soon as possible after hospital admission, and always within four days. The MoCA was performed at any time during the stay at the stroke unit, according to clinical routine. Details regarding the individual’s current smoking status and information on whether or not the individual used a walking aid while at the stroke unit were collected. Systolic blood pressure was measured in supine position after at least 5 min of rest. Information regarding the individual’s current smoking status and comorbidities was collected by reviewing the participants’ medical records.

### Assessment of the dependent variable at 6 months after stroke

**Figure 1:**
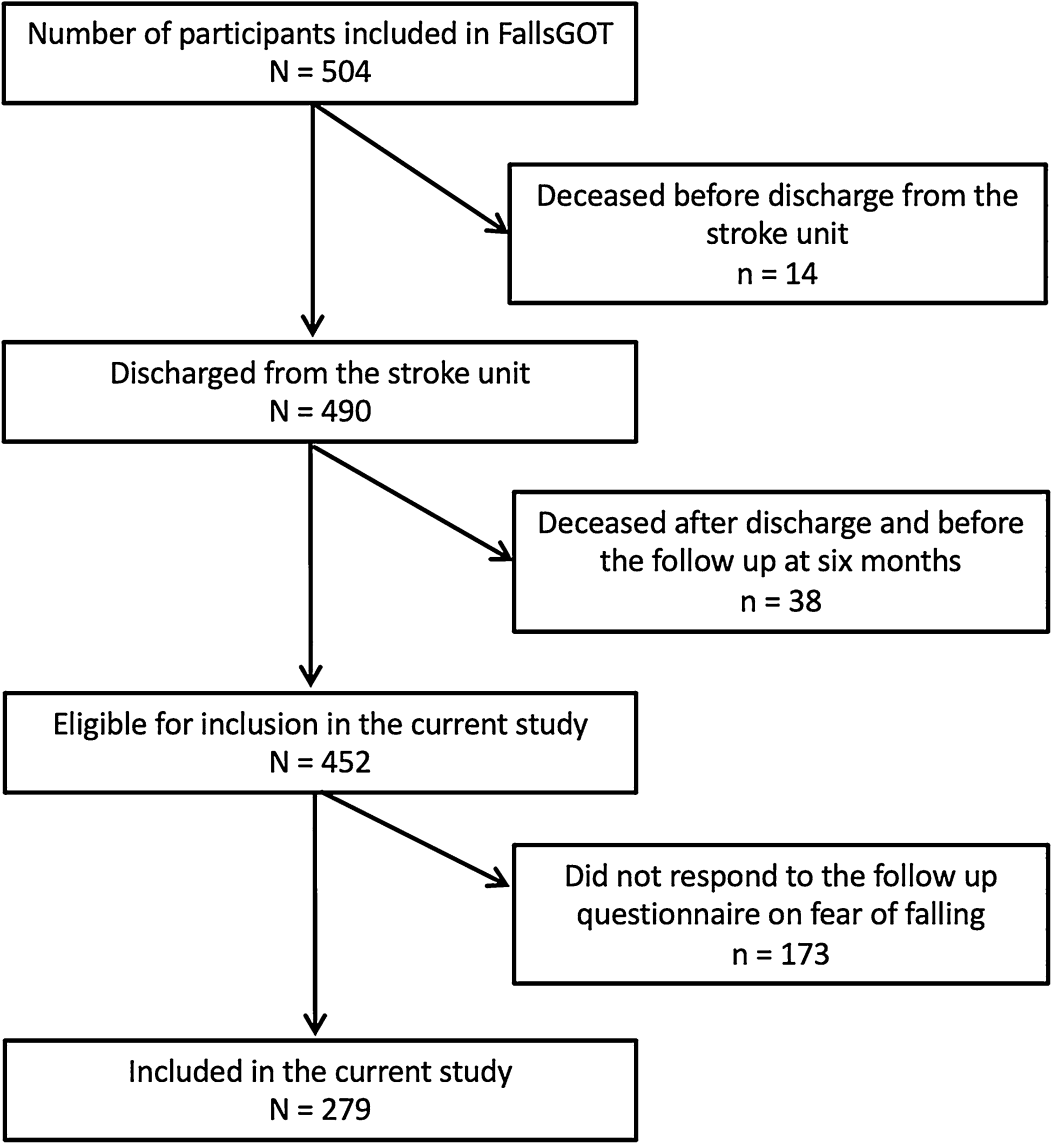
Flowchart of the inclusion process.

The dependent variable, fear of falling at 6 months after stroke, was assessed using a questionnaire containing the question, ‘Are you afraid of falling?’ with “yes” and “no” as possible answers. The questionnaire was sent by post at the same time as the request for participation. All the participants received the questionnaire within a time span of 14 days before or after completing 6 months after stroke. Everyone who answered this questionnaire was included in the current study. The inclusion process of the study is shown in Fig. 1.

### Statistical analyses

The statistical analyses were performed using the IBM Statistical Package for Social Sciences software, version 24. Descriptive statistics, such as means, medians, standard deviations and interquartile ranges, were presented. Logistic regression was used to identify the factors that were associated with the dependent variable, presence of fear of falling at 6 months after stroke. The independent variables that were analysed were: age; sex; height; weight; location of stroke; previous fall(s) at the stroke unit; length of stay at the stroke unit; use of a walking aid; being a smoker/non-smoker; stroke severity using the NIHSS score; postural control using the SwePASS score and self-reported physical activity level using the SGPALS. Multicollinearity between the independent variables was determined using Spearman’s rank correlation, with correlation coefficients of ≥0.7 indicating multicollinearity^43^.

Univariable logistic regression was performed first, after which variables with *p*-values of < 0.1 were included in the multivariable logistic regression model. In the multivariable regression model, a *p*-value of < 0.05 was considered to be statistically significant. The goodness-of-fit for the multivariable logistic model was tested using the Hosmer–Lemeshow test and the improvement in fit was verified using Cox and Snell as well as Nagelkerke pseudo R^2^ values. The results of the logistic regression models were presented as odds ratios (OR), 95% confidence intervals (CI) and *p*-values. The area under the receiver operating characteristic (ROC) curve was presented for each variable and for the final prediction model. An area of 70–79% under the curve was regarded as acceptable, 80–89% was regarded as excellent and 90–100% was regarded as outstanding diagnostic accuracy^44^.

### Ethical declarations

Ethical approval was obtained from the Swedish Ethical Review Authority, Gothenburg, Sweden (No. 004–14 February 25, 2014 and No. T301-15, Ad 004 -14 April 9, 2015). Written informed consent was obtained from all participants or next of kin prior to their inclusion in the study. All methods were performed in accordance with the relevant guidelines and regulations of the Swedish Ethical Review Authority. The FallsGOT study was listed in ClinicalTrials.gov with trial registration number NCT02264470.

## Results

A total of 279 individuals from the FallsGOT cohort responded to the questionnaire and were included in this study. The demographic and baseline characteristics of the study group are presented in Table 1. Descriptive statistical analysis showed that 117 (42%) of the participants had fear of falling at 6 months after stroke. The mean age of the participants was 76 years and a small majority of the participants were women. One in ten participants had experienced a fall while at the stroke unit. The median NIHSS score was 1, indicating that a large proportion of the patients had had a minor stroke. The SwePASS median score was three scores lower for those who experienced fear of falling compared with those who did not. The majority of the participants (51%) reported being physically inactive prior to the stroke onset. None of the participants reported high physical activity level by marking the response category of 4 in the SGPALS.

**Table 1:** Demographic characteristics at baseline for all participants and for those that reported having or not having fear of falling.

Characteristics [mean ± SD, median (IQR), n (%)]	All participants (n = 279)	Fear of falling at 6 months after stroke (n = 117)	No fear of falling at 6 months after stroke (n = 162)
Age (years)	75. 83 ± 11.17	78.05 ± 11.13	74.22 ± 10.95
Women Men	143 (51.3) 136 (48.7)	71 (60.7) 46 (39.3)	72 (44.4) 90 (55.6)
Height (cm) (missing, n = 3)	169 (162–177)	167 (160–176)	171 (163–179)
Weight (kg) (missing, n = 1)	75 (63–86)	74 (62–85)	75 (64–87)
**Location of stroke lesion, hemisphere**			
Right	148 (53.1)	64 (54.7)	84 (51.9)
Left	115 (41.2)	48 (41.0)	67 (41.4)
Bilateral	7 (2.5)	1 (0.9)	6 (3.7)
Unknown	9 (3.2)	4 (3.4)	5 (3.0)
**Type of stroke**			
Infarction	261 (93.5)	109 (93.2)	152 (93.8)
Haemorrhagic	16 (5.7)	8 (6.8)	8 (4.9)
Subarachnoid haemorrhage	2 (0.8)	0 (0)	2 (1.3)
Length of stay at the hospital (days)	8 (4–14)	8 (5–14.5)	7 (4–13)
**Oxfordshire system of stroke classification (missing, n = 1)**			
Total anterior circulation	4 (1.6)	1 (0.8)	3 (2.1)
Partial anterior circulation	81 (29.2)	37 (31.6)	44 (27.3)
Lacunar stroke	102 (36.6)	47 (40.2)	55 (34.2)
Posterior circulation	91 (32.6)	32 (27.4)	59 (36.4)
Falls at the stroke unit (yes)	30 (10.8)	18 (15.4)	12 (7.4)
NIHSS median score (missing, n = 21)	1 (0–3)	2 (0–4)	2 (0–3)
MoCA median score	25 (22–27)	25 (21–27)	26 (23–28)
**Fear of falling during the acute phase of stroke (missing, n = 8)**			
Yes	126 (46.5)	80 (70.2)	46 (29.3)
No	145 (53.5)	34 (29.8)	111 (70.7)
SwePASS median score (missing, n = 3)	30 (22–34)	28 (22–30)	31 (29–34)
**SGPALS total score**			
1—physically inactive	142 (51.1)	77 (66)	65 (40)
2—some physical activity	121 (43.2)	38 (33)	83 (51)
3—regular physical activity	16 (5.7)	2 (1)	14 (9)
Current smoker (missing, n = 9)	53 (19.6)	23 (19.7)	30 (18.5)
Use of a walking aid (yes)	116 (41.6)	64 (54.7)	52 (32.1)
Systolic BP on fourth day after stroke (mm Hg) (missing, n = 91)	142 (130–157)	140 (99–210)	140 (125–156)
Diabetes mellitus	59 (21.1)	31 (26.5)	28 (17.3)
Hypertension	213 (76.4)	97 (82.9)	116 (71.6%)
Congestive heart failure	24 (8.6)	9 (7.7)	15 (9.3)
Ischemic heart disease	61 (21.9)	27 (23.1)	34 (21)
Atrial fibrillation	74 (26.5)	28 (23.9)	46 (28.4)
Previous TIA	28 (10)	9 (7.7)	19 (11.7)
Previous stroke	85 (30.5)	46 (39.3)	39 (24.1)

BP, blood pressure; IQR, interquartile range; MoCA, Montreal Cognitive Assessment; SGPALS, Saltin-Grimby Physical Activity Level Scale; SwePASS, the Modified Version of the Postural Assessment Scale for Stroke Patients; TIA, Transient Ischemic Attack.

No multi-collinearity was found, except between sex and weight where male sex was correlated with higher weight (correlation coefficient = 0.70). The results of the univariable and the multivariable logistic regression analyses of the independent variables against the presence of fear of falling as the dependent variable are shown in Table 2. Univariable regression analysis revealed that age, sex, height, fall(s) at the stroke unit, the use of a walking aid, SwePASS score and SGPALS score were significantly associated with fear of falling at 6 months after stroke.

**Table 2:** Univariable and multivariable analyses for prediction of fear of falling 6 months after stroke.

Predictor	Univariable analysis		The area under the ROC curve (%)	Multivariable analysis of the significant variables in univariable analysis	
	Odds ratio (95% CI)	*P*-value		Odds ratio (95% CI)	*P*-value
Age	1.03 (1.0–1.06)	**0.005**	60	1.00 (0.97–1.02)	0.89
**Sex**					
Male (ref.)	1				
Female	1.93 (1.19–3.12)	**0.008**	58	1.84 (0.90–3.80)	0.10
Height	0.98 (0.95–1.00)	**0.046**	43	1.00 (0.97–1.04)	0.93
Weight	0.99 (0.98–1.01)	0.736	47		
**Location of stroke**			48		
Left (ref.)	1				
Right	0.95 (0.25–0.37)	0.94			
Bilateral	0.89 (0.23–3.5)	0.87			
Unknown	0.21 (0.01–2.52)	0.22			
**Fall(s) at the stroke unit**					
Yes (ref.)	2.27 (1.05–5.04)	**0.037**	54	1.92 (0.76–5.04)	0.16
No (ref.)	1				
Length of stay at the stroke unit (days)	1.01 (0.99–1.04)	0.30	58		
**Use of walking aid**			61		
Yes	0.39 (0.24–0.64)	** < 0.001**		0.81 (0.44–1.48)	0.50
No (ref.)	1				
**Current smoker**	1.08 (0.59–1.98)	0.799	51		
No	1				
**NIHSS score**			52		
Mild (0–4) (ref.)	1				
Moderate (5–15)	0.67 (0.04–10.9)	0.781			
Severe (16–42)	0.94 (0.05–16.3)	0.969			
**SwePASS score (postural control)**			67		
Good (31–36) (ref.)	1				
Moderate (25–30)	4.95 (2.52–9.75)	** < 0.001**		2.32 (0.98–5.52)	0.06
Poor (0–24)	3.86 (2.14–6.96)	** < 0.001**		**2.60 (1.26–5.36)**	**0.01**
**SGPALS score**					
Physically active (2–4) (ref.)	1				
Physically inactive (1)	2.87 (1.75–4.71)	** < 0.001**	63	**2.04 (1.01–4.12)**	**0.048**

CI, confidence interval; ROC, Receiver Operating Characteristic; SwePASS, The Modified Version of the Postural Assessment Scale for Stroke Patients; SGPALS, the Saltin-Grimby Physical Activity Level Scale. Variables with p-values less than 0.1 and 0.05 were considered significant for the univariable and multivariable analyses respectively, and are presented in bold. For the multivariable regression model, the *p*-value of the Hosmer and Lemeshow test was 0.31, the Cox and Snell R^2^ was 0.14 and the Nagelkerke R^2^ was 0.19. Included in the multivariable analysis, n = 276.

The final model was obtained using multivariable logistic regression analysis, where poor postural control (a SwePASS score of ≤ 24) and being physically inactive (SGPALS score = 1) remained as statistically significant predictors of fear of falling. Individuals with poor postural control after an acute stroke had a 2.6 times higher odds ratio for fear of falling at 6 months after stroke compared with those with good postural control (a SwePASS score of 31–36). Additionally, individuals that reported being physically inactive prior to stroke had a two times higher odds ratio for fear of falling 6 months after stroke compared with those that reported a light to high physical activity level. The accuracy of the final model was estimated to be 71% using the area under the ROC curve. Table 3 shows the probability of fear of falling related to the presence or absence of the statistically significant predictors obtained from the multivariable analysis. Among those with poor or moderate postural control in the acute phase and low physical activity prior to the stroke, 61% experienced fear of falling after 6 months, compared with 20.5% of those with good postural control, who were physically active.

**Table 3:** Probability of fear of falling related to the presence or absence of risk factors (N = 279).

Parameter	Probability of fear of falling, n/N (%)
Physically active and good postural control	17/83 (20.5)
Physically active and moderate postural control	17/42 (40.5)
Physically active and poor postural control	6/12 (50.0)
Physically inactive and good postural control	8/28 (28.6)
Physically inactive and moderate postural control	38/62 (61.3)
Physically inactive and poor postural control	30/49 (61.2)

Physically active: SGPALS score 2–3; physically inactive: SGPALS score 1; good postural control: SwePASS score ≥ 31; moderate postural control: SwePASS score 25–30; poor postural control: SwePASS score ≤ 24.

## Discussion

In a sample of 279 individuals, we identified the incidence of fear of falling using a single question and determined the factors prior to and in the acute phase after a stroke that are associated with fear of falling at 6 months after stroke. The results showed that 42% of the individuals experience fear of falling at 6 months after stroke. We found that poor postural control and self-reported physical inactivity prior to the stroke were the predictors of fear of falling at 6 months after a stroke event. Our hypothesis that poor postural control and self-reported physical inactivity were associated with fear of falling was confirmed. However, our hypothesis that female sex, the use of a walking aid and fall(s) at the stroke unit were associated with fear of falling was rejected. The multivariable model built as a part of this study had acceptable diagnostic accuracy. The probability of fear of falling varies greatly depending on the presence or absence of risk factors. In physically inactive individuals with poor or moderate postural control just over 13 of 20 reported fear of falling compared with eight of 20 in physically active individuals with good postural control. To our knowledge, this is the first study with a relatively large sample size that shows the association between fear of falling at 6 months after stroke using assessments performed in the acute phase after a stroke.

The proportion of individuals who reported fear of falling (42%) in the current study is in line with previous research conducted in the late subacute phase of stroke where the proportion was between 32% and 50%^7,18,26^. When compared with another study performed in the same cohort of individuals, fear of falling at 6 months after stroke was slightly reduced in the current study (42%) compared with the acute phase of stroke (51%)^21^.

Our finding that poor postural control is one of the significant predictors of fear of falling is in line with a previous study with the same cohort as the current study, which showed that poor postural control was a significant predictor of fear of falling in the acute phase of stroke^21^. This finding also corroborates a study performed in the chronic phase of stroke which showed that people with fear of falling have poorer postural control when standing and walking^23^. Poor postural control has also recently been shown to be associated with falls in the first year after stroke^28,35^. Considering these findings together, it can be assumed that postural control is one of the strongest predictors of both risk of falling and fear of falling after stroke^21,28,35^. Given that the time taken for a SwePASS assessment is approximately 8 min^38^, measuring postural control in the acute phase after a stroke is probably the fastest and most efficient method for predicting both fear of falling and risk of falling after stroke.

The finding that self-reported physical inactivity is a predictor of fear of falling matches research in the chronic phase of stroke, where factors such as higher sitting time, less functional mobility, reduced activity and low participation, all of which lead to physical inactivity, were found to be associated with greater fear of falling^12,23–25^. The validity of our finding is also strengthened by a research finding on reverse association, where physical activity such as walking has been shown to reduce fear of falling in individuals after stroke^45^. It is possible that individuals with less prior physical activity are less confident about their walking ability, have poorer muscle strength, reduced co-ordination, lower postural equilibrium, longer reaction times and consequently more fear of falling after stroke. Furthermore, the finding is in agreement with systematic reviews that have found that physical activity is likely to reduce the risk and fear of falling to a moderate degree in older adults^46,47^. Given that physical inactivity is a risk factor for stroke^48^, it is important to provide targeted interventions to promote physical activity prior to stroke in order to reduce the incidence of fear of falling after stroke, as well as stroke itself.

In the acute phase after a stroke, females reported fear of falling more frequently than men^21^. Our study found that this holds true at 6 months after stroke, and that female sex was a predictor of fear of falling in the univariable analysis, but not in the multivariable analysis. Similarly, another study conducted at 12 months after stroke also showed that female sex was a predictor of fear of falling in the univariable analysis^49^. We also found that those who did not use a walking aid at the stroke unit reported more fear of falling at 6 months after stroke, in contrast to the results of a previous study performed during the acute phase of a stroke^21^. The reasons for the differences in associations between fear of falling and female sex as well as using a walking aid, at the different time points are unclear. The association between fear of falling and a history of previous falls has been quantified in the chronic phase of stroke^24^, and has been described qualitatively in the subacute phase of stroke^7^, but the current study was not able to find any such association at 6 months after stroke.

The results of this study may help us to identify the individuals at risk of fear of falling at 6 months after stroke. As the high-risk individuals could be identified as early as during the first week after hospital admission after stroke, subsequent early assessments and interventions may be provided to them to reduce fear of falling, thereby increasing quality of life. Interventions such as physical exercise in the form of walking^45^, cognitive behavioural therapy^50^ and balance training^50^, could be used to reduce fear of falling in individuals with stroke. Based on the findings in the current study, further research to gather evidence with a view to developing interventions that are effective in reducing fear of falling after stroke is warranted.

The strength of this study is the relatively large sample of individuals with stroke. We believe that these results could be applied to individuals in the acute and subacute phase, mainly after a mild stroke. The predictors identified in this study were easy to measure and therefore, less time consuming to collect. This study, however, is not without limitations. Of the 452 individuals still alive 6 months after stroke, only 279 were included in the current study, which might have caused a selection bias. We have no information on fear of falling in 38% of individuals who did not respond to the questionnaire. A further selection bias might have been caused by not including those who underwent a thrombolysis or thrombectomy. The question about fear of falling has not been tested for reliability or validity. There could also be additional predictors of fear of falling, which has not been considered in this study. Further research could also include qualitative approach for a deeper understanding of the predictors of fear of falling. As in all observational studies, the cause and effect relationship between the dependent and independent variables cannot be determined. The clinical implication of this study is that the identification of individuals at risk of fear of falling at 6 months after stroke can be achieved by one single question about physical activity level prior to stroke along with an easily performed assessment of postural control in the acute phase of stroke.

## Conclusion

The findings in this study show that physical inactivity prior to stroke and poor postural control in the acute phase after a stroke and are associated with fear of falling 6 months after stroke, which could be addressed using population-based targeted interventions prior to stroke and in the individualised rehabilitation plans early after stroke.

## Data Availability

The dataset is available from the principal investigator, Carina U. Persson (carina.persson@vgregion.se), in response to a reasonable request. According to Swedish regulations, permission to use data can be obtained after an application to and approval by the ethics committee.
